# Laboratory Selection and Assessment of Resistance Risk in *Drosophila suzukii* (Diptera: Drosophilidae) to Spinosad and Malathion

**DOI:** 10.3390/insects12090794

**Published:** 2021-09-04

**Authors:** Joseph Onwusemu Disi, Ashfaq A. Sial

**Affiliations:** Department of Entomology, University of Georgia, Athens, GA 30302, USA; JOSEPH.DISI@uga.edu

**Keywords:** spotted-wing drosophila, spinosyns, organophosphate, dose–response bioassays, resistance development, realized heritability

## Abstract

**Simple Summary:**

Continuous insecticide applications used to prevent fruit infestations by spotted-wing drosophila (SWD), an invasive pest of soft-skinned fruits worldwide, can elevate the risk of resistance development in *D. Suzukii* field populations. However, proactive assessment of resistance risk using laboratory selection provides valuable information for development of sustainable resistance management strategies for SWD. After 10 and 11 generations of artificial selection of a colony of field-collected SWD for resistance against spinosad and malathion, a 7.55- and 2.23-fold resistance to spinosad and malathion was realized. A quantitative genetic approach used to estimate realized heritability (*h*^2^) of resistance shows that the risk of resistance in SWD populations exists against both spinosad and malathion, and a faster rate of resistance development is expected against spinosad. However, timely implementation of resistance management strategies can slow the development of resistance and prolong effective life of these insecticides against *D. suzukii*.

**Abstract:**

*Drosophila suzukii* (Matsumura) is one of the most economically important pests of soft-skinned fruits worldwide. Repeated insecticide applications commonly used to prevent fruit infestations increase the risk of resistance development in *D. suzukii*. Assessment of resistance risk in *D. suzukii* using artificial selection can be valuable in developing proactive resistance management strategies to retain susceptibility in the field populations. Here, we artificially selected a colony of field-collected *D. suzukii* for resistance against spinosad and malathion. A quantitative genetic approach was then used to estimate realized heritability (*h*^2^) of resistance and predict the rates of resistance development. After 10 and 11 generations of selection, resistance to spinosad and malathion in *D. suzukii* females significantly increased by 7.55- and 2.23-fold, respectively. Based on the predicted rates of resistance development, assuming *h*^2^ = 0.14 (mean *h*^2^ of spinosad resistance in this study) and 90% of population was killed at each generation, 10-fold increase in LC_50_ of *D. suzukii* females would be expected in nine generations for spinosad. However, 10-fold increase in LC_50_ of *D. suzukii* females for malathion would be expected in 37 generations, assuming *h*^2^ = 0.08 (mean *h*^2^ of malathion resistance) and 90% of population was killed at each generation. These results indicate that the risk of resistance in *D. suzukii* populations exists against both spinosad and malathion. However, resistance would develop faster against spinosad as compared to malathion. Thus, resistance management strategies should be implemented proactively to maintain the effectiveness of these insecticides to control *D. suzukii*.

## 1. Introduction

*Drosophila suzukii* Matsumura (Diptera: Drosophilidae), commonly referred to as spotted-wing drosophila (SWD), has emerged as an economically important insect pest of soft-skinned fruits in many parts of the world including Europe, North America and Africa [1,2,3,4]. Female *D. suzukii* selectively lay eggs in ripening and ripe fruits where early instars complete their development within the fruit rendering the fruits unmarketable. Economic impact of this adaptive behavior of *D. suzukii* females in the berry industry is estimated to be over 421.5 million US dollar per year in worst-case infestation years [2]. In addition, farmers face significant economic losses if fruits are rejected in export sorting facilities because of detection of larvae in the fruits. 

Control options tested or used for management of *D. suzukii* in places where economic pressures are found are mechanical exclusion [5], management of crop microclimate [6], and behavioral modifications [7,8,9]. However, due to extremely low tolerance for infested fruit in the market, preventative insecticide applications are the primary means to control *D. suzukii* [10,11,12]. Spinosad (spinosyn) and malathion (organophosphate) are among the two most used insecticides for control of *D. suzukii* [10,11,12]. While conventional fruit farmers may have an array of insecticides to choose from in addition to malathion [13], organic producers are limited to spinosad, being the only effective OMRI (Organic Materials Research Institute) approved insecticide for use in organic blueberry production. Malathion is widely used in *D. suzukii* control programs in blueberries because of its effectiveness and a very short pre-harvest interval of one day [3,10,11,12]. Therefore, berry fruit farmers make multiple applications of spinosad and malathion year in and year out, which increases the potential for resistance development in *D. suzukii* field populations. A recent study has already reported significant levels of resistance to spinosad in *D. suzukii* field populations collected from organic fruit farms in California [14]. In contrast, *D. suzukii* susceptibility to malathion was unaffected in a laboratory colony in British Columbia, Canada even after several generations of continuous exposure to malathion [15]. However, once developed, the ecological backlash of insecticide resistance can be costly due to the potential for control failures [16,17,18,19,20,21,22]. Armes et al. [16] reported huge control failures in India’s cotton belt due to *Helicoverpa armigera* resistance to insecticides. 

It is therefore important to assess resistance risk using flies collected from fields where these insecticides are frequently used against *D. suzukii*. Risk assessment will provide information on current resistance status and genetic basis of resistance, which is critical to develop integrated resistance management (IRM) strategies. Farmers will then be able to implement those strategies proactively to slow the development of resistance and continue to benefit from pest management products. The goal of this study was to assess the risk of resistance development to spinosad and malathion in *D. suzukii* populations to ensure that the products are effective in controlling *D. suzukii*. Specific objectives pursued were to: (1) artificially select *D. suzukii* for resistance to spinosad and malathion and (2) estimate realized heritability and predict rates of resistance development in *D. suzukii* against spinosad and malathion. 

## 2. Materials and Methods

### 2.1. Insects

A total of five field populations of *D. suzukii* were collected after the 2017 field season’s harvest from blueberry plantations located in four counties in Georgia, USA. Briefly, apple cider vinegar dispensed with paper towel in modified 32 oz plastic cup traps were placed on the border rows in blueberry fields. Traps were retrieved three days later, and live captured flies were aspirated and identified under the microscope in the laboratory. One of these populations was from an organic blueberry farm while the other four were collected from conventional commercial blueberry farms without prior history of spinosyns or malathion resistance [23]. To have maximum genetic variation after maintaining the colonies for two generations in the laboratory, 50 mated females from each of the five field populations were used to develop a mixed population. Flies from the mixed populations were used for the insecticide resistance selection in the laboratory. The *D. suzukii* colonies were reared on a standard insecticide free cornmeal media [24] in growth chamber at 24 ± 1 °C, 65 ± 5% RH and 14:10 h (L: D) photoperiod. 

### 2.2. Selection of Drosophila suzukii Resistance to Spinosad and Malathion

Selection was performed using glass vial method developed for adult *D. suzukii* contact bioassays with slight modifications [23,25]. The insecticides used were spinosad (Entrust^TM^ 24 SC, Dow AgrowSciences LLC, Indianapolis, IN, USA) and malathion (Malathion 8F, Gowan Company LLC, Yuma, AZ, USA). Acetone was used to dissolve malathion while formulated spinosad was dissolved in ddH_2_O and 1266.6 µL L^−1^ of an adjuvant (Induce^TM^ brand, Helena Chemical Company, Memphis, TN, USA). The concentrations used for the selections were 8.15 ppm for malathion and 21.6–38.02 ppm for spinosad and have been shown to cause nearly 100% mortality in susceptible flies from a laboratory colony. These concentrations were developed from a series of preliminary laboratory assays conducted to optimize the rapid assessment protocol for detection of insecticide resistance (RAPID) assays previously reported in Van Timmeren et al. [23] and Gress and Zalom [14]. For malathion, 2 mL of insecticide was pipetted into a 500 mL glass bottle, whirled for about 30 s to coat inside surface of the bottle. The glass bottle and the content were placed on a hotdog roller for about 10 min to dry. On the other hand, formulated spinosad was dissolved in a water-induce mixture, a 2 mL measure was pipetted into a 500 mL bottle and whirled as described above. Unlike the bottles that were coated with malathion, heat was applied for 30 min to hasten the drying of spinosad-coated bottles. The next morning, 10 male and 10 female flies, 3–7 days old, were aspirated into 20 mL scintillation vials without insecticides in preparation to being released into the insecticides-treated bottles for maximum exposure. A total of 7000 flies (1:1 sex ratio) was released into thirty-five 500 mL insecticide-coated glass bottles without diet (i.e., 200 per bottle) in the beginning of the selection. Flies that survived after 3 h of exposure to malathion or 6 h of exposure to spinosad were transferred to plastic drosophila rearing bottles without insecticides, but with a thin layer of media to provide moisture. After 24 h, dead flies were counted while survivors were moved to freshly made media without insecticide where they were allowed to reproduce. Offspring were again exposed in the insecticide treated bottles as described above. This process was repeated for a total of 10 rounds of selection for spinosad and 11 rounds of selection for malathion. Percentage of survivors were determined for every round of selection from the offspring.

### 2.3. Dose–Response Bioassay

Following previously developed *D. suzukii* susceptibility bioassay protocol with modifications, dose–response bioassays were performed with spinosad- and malathion-selected fly strains, and flies from the unselected mixed population were used as a reference susceptible population to determine any changes in susceptibility of the flies due to selection. Depending on the availability of flies, dose–response bioassays were performed for F_3_, F_8_ and F_10_ in the case of spinosad and F_7_, F_9_ and F_11_ in the case of malathion. A total of 6–8 concentrations of each insecticide were used to conduct dose–response bioassays, which ranged from 3 to 15 ppm for malathion and 3–300 ppm for spinosad. Briefly, 1 mL of insecticide was pipetted into the 20 mL scintillation vials, whirled for about 30 s to coat the inside surface of the vial, after which the content was dried as previously described for the selection bioassay. Five male and five female *D. suzukii* flies were first aspirated into insecticide-free 20 mL scintillation vials and gently released into the insecticide-treated vials. Mortality was assessed after 3 h of exposure to malathion and 6 h of exposure to spinosad in vials coated with malathion or spinosad. Live, moribund, or dead flies were counted. Moribund in this study was defined as inability of the flies to right themselves on their legs in 30 s after being flipped on their nota in scintillation vial. Moribund flies were recorded as dead for data analysis. The experiment was replicated three times per dilution concentration for a total of 90–105 flies per dose response bioassay.

### 2.4. Data Analysis

Mortality data were subjected to probit analysis using Polo plus (LeOra software) to determine median lethal concentrations (LC_50_) values along with their corresponding 95% fiducial limits. The resistance ratio and their 95% confidence limits were calculated by dividing the LC_50_ of the selected strains of *D. suzukii* by the LC_50_ of the unselected mixed population. Resistance ratio was considered significantly different if the 95% confidence limits generated with mortality data from the selected strain did not overlap with that of the unselected mixed population. Unequal selection of males and females was observed in this study, and as a result separate estimation was made for both sexes throughout the paper to minimize bias that may be introduced by pooling mortality data between males and females.

### 2.5. Estimation of Realized Heritability

Realized heritability (*h*^2^) of *D. suzukii* selected for resistance to spinosad and malathion in the laboratory was calculated using the threshold trait analysis method developed by Tabashnik [26]. Thus, realized heritability (*h*^2^) = response to selection (*R*)/selection differential (*S*).

Response to selection (*R*) was calculated as:

*R* = log (final LC_50_) − Log (initial LC_50_)/*n*,

where final LC_50_ is the LC_50_ computed after *n* generations of selection for resistance to spinosad or malathion in this study and initial LC_50_ is the LC_50_ of the unselected mixed population (parental population) before selection. 

The selection differential (S) was calculated as:

S = *iσ_p_*

where *i* is the intensity of selection and *σ_p_* is the phenotypic standard deviation. 

According to Tabashnik and McGaughey [27], selection intensity can be estimated using the equation:

*i* = 1.583 − 0.0193336 *p* + 0.0000428 *p*^2^ + 3.65194/*p*,

where *p* is the percent survivorship after selection with spinosad or malathion. 

The phenotypic standard deviation (*σ_p_*) was estimated as:

*σ_p_* = [1/2 (initial slope + final slope)]^−1^,

where initial and final slope values are those obtained from probit regression lines of parental population before selection and the offspring after *n* generations of selection with spinosad or malathion.

### 2.6. Projection of Number of Generations for 10-Fold Increase in Resistance Development

The response of *D. suzukii* to selection in the laboratory can be used to predict risk of resistance development in terms of how many generations it may take for a 10-fold increase in resistance to the target pest control product. These quantitative genetics approach allows for estimation of potential control efficacy of an insecticide given the rate at which realized heritability increases or decreases during artificial selection in the laboratory [28]. Thus, by varying heritability and slope at different selection intensities, the number of generations (*G*) required for a 10-fold increase in resistance in *D. suzukii* selected to spinosad or malathion was calculated as the reciprocal of *R*:

G = R^−1^ = (*h*^2^*S*)^−1^

The equation Q = R/*i* was used to estimate the response quotient (Q) necessary for comparing rates of resistance development against spinosad and malathion without reference to slope, which is not constant among insecticides [26]. Q is valuable in evaluating the durability of any insecticide against any given pest population.

## 3. Results

### 3.1. Selection of Drosophila suzukii Resistance to Spinosad and Malathion and Dose–Response Bioassay

As a result of repeated exposure to spinosad or malathion over several generations, the susceptibility of the selected populations to the respective insecticides significantly decreased over time (Table 1 and Table 2). Overall, the LC_50_ of female *D. suzukii* for the tested insecticides, spinosad and malathion, was higher than that of male *D. suzukii* (Table 1 and Table 2). The LC_50_ of spinosad-selected females was 50.72 and 65.73 ppm in the F_3_ and F_8_ selected generations and then increased significantly to 167.25 ppm in the F_10_ generation compared to the unselected mixed population females, thus representing a 7.55-fold increase in LC_50_ (Table 1). Although the LC_50_ was generally lower for male flies compare with the females, a similar increasing trend in LC_50_ was observed in the tested generations with LC_50_ of 105.79 ppm recorded in the F_10_ male *D. suzukii*. 

For malathion-selected population, LC_50_ of malathion-selected females was recorded as 3.72 ppm after 7 generations of selection which significantly increased to 7.81 ppm after 11 generations of selection representing a 2.23-fold increase in LC_50_ (Table 2). Likewise, LC_50_ of malathion-selected males was recorded as 3.23 ppm after 7 generations of selection which significantly increased to 5.49 ppm after 11 generations of selection. 

### 3.2. Estimation of Realized Heritability

**Figure 1:**
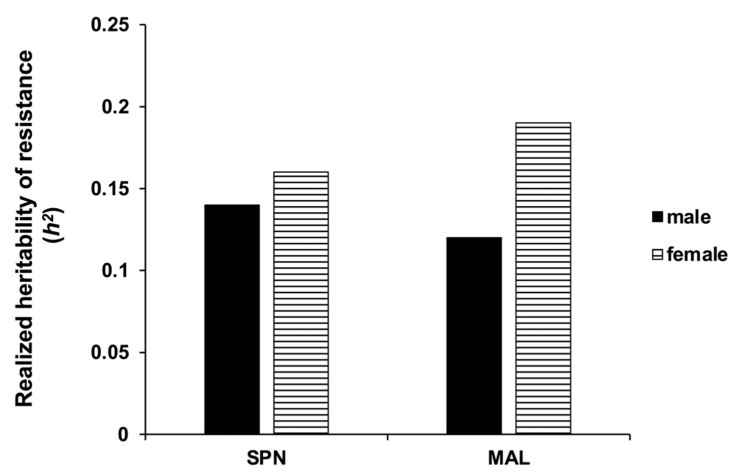
Realized heritability (*h*^2^) of resistance to spinosad and malathion in adult *D. suzukii* populations selected for resistance in the laboratory.

After 10 generations of selection, realized heritability (*h*^2^) of spinosad resistance in female *D. suzukii* of the spinosad-selected population was estimated at 0.16 and *h*^2^ of spinosad resistance in male *D. suzukii* was 0.14. Similarly, after 11 generations of selection, *h*^2^ of malathion resistance in female *D. suzukii* was estimated at 0.12 and *h*^2^ of malathion resistance in male *D. suzukii* was 0.20. Overall, *h*^2^ of malathion resistance was slightly higher than *h*^2^ of spinosad resistance in female *D. suzukii* of the selected populations, and vice versa in male *D. suzukii* (Figure 1).

Over the course of these selection experiments, the mean values of response to selection (R) were 0.08 (female) and 0.05 (male), and the mean values of selection differential (S) were 0.67 (female) and 0.65 (male) for spinosad selection (Table 3). For malathion selection, the mean value of R was 0.01 (both female and male), while mean values of S were 0.21 (female) and 0.29 (male) (Table 4). 

**Figure 2:**
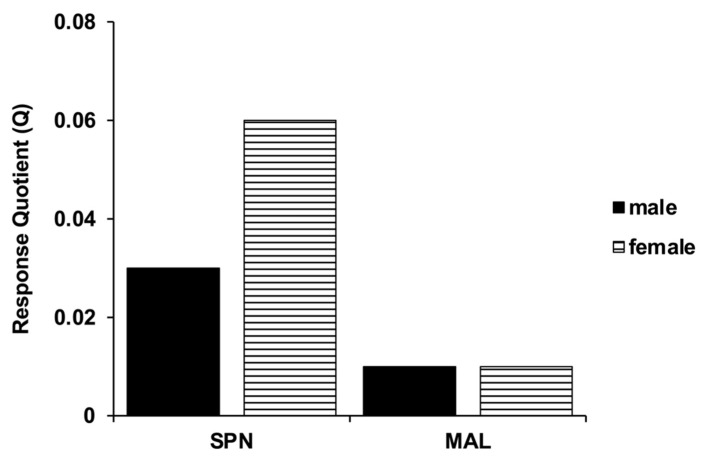
Response quotient (Q) comparing rates of resistance against spinosad and malathion in adult *D. suzukii* populations selected for resistance in the laboratory.

Overall, the response to selection (R) for malathion was lower as compared to the R for spinosad. The mean values of Q for malathion resistance in both male and female (0.01) were also lower than those for spinosad resistance in female (0.06) and male (0.03) *D. suzukii* (Figure 2). 

## 4. Discussion

The significant decline in susceptibility of *D. suzukii* adults to spinosad and malathion, the two most used insecticides for fruits and vegetables in the United States, in this study indicates that resistance genes are present in the field populations that were used to establish the mixed colony even though no *D. suzukii* control failures associated with resistance have been reported yet [23]. Furthermore, the fact that the response quotient was higher for spinosad than malathion in the mixed field population in this study indicates that the resistance would develop at a faster rate against spinosad as compared to malathion. 

This is the first report of *D. suzukii* adults’ resistance to malathion in a laboratory selection study. After eleven generations of mass selection under high malathion intensity, susceptibility of malathion-selected *D. suzukii* adults decreased 2.23-fold compared to the unselected population. This finding corroborates previous report that showed that *D. melanogaster* resistance tended to increase more rapidly in high malathion selection intensity [29]. High selection intensity can eliminate unwanted alleles in selected populations even though use of high malathion concentration during selection bioassays has been reported to reduce genetic diversity due to very low survival rate [30,31]. Although we experienced high fly mortalities due to exposures to malathion in this study, we believe that the significant level of resistance obtained after only eleven generations of selection was due to genetic variation that was present in the population used to initiate the selection study. In contrast to our findings, the use of an inbred laboratory line of *D. suzukii* with little genetic variation did not permit selection of high malathion resistance even after 30 generations [15]. Our findings, though significant, do not represent a potential control failure with malathion. Rather, they serve as a caution that malathion resistance can emerge in field populations if proper IRM programs are not implemented.

Realized heritability estimates are important predictive indices to assess risk of resistance development in situations where there is limited information about the genetic basis of resistance [26]. In our study, the realized heritability (*h*^2^) values of 0.2 and 0.16 indicate that 20% of the variation in susceptibility of the selected population to malathion compared to 16% of the variation in susceptibility of the selected population to spinosad was caused by additive genetic variance in females. Even though, *h*^2^ of malathion resistance was slightly higher than spinosad resistance in the female *D. suzukii* of the selected population, the majority of genetic variation in susceptibility to malathion was selected out of the population during the initial few rounds of selection due to high mortality. Consequently, the level of resistance to malathion was lower as compared to spinosad resistance in this study.

**Figure 3:**
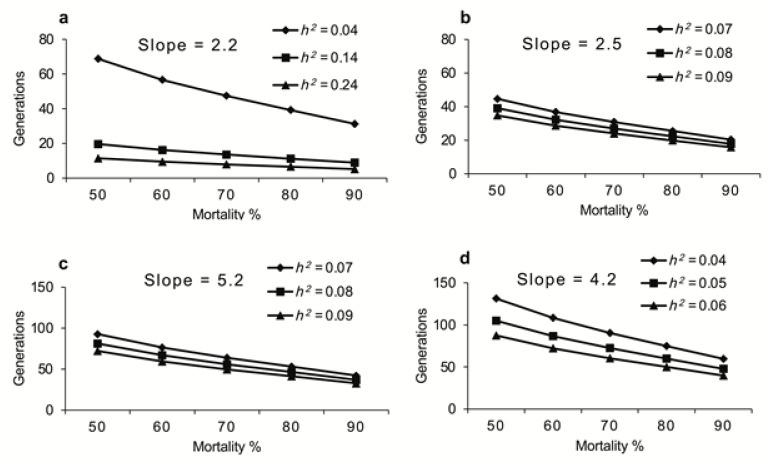
Effect of realized heritability on number of generations of *D. suzukii* required for a 10-fold increase in LC_50_ of female exposed to spinosad (**a**), male exposed to spinosad (**b**), female exposed to malathion (**c**) and male exposed to malathion (**d**) at different selection intensities (i).

**Figure 4:**
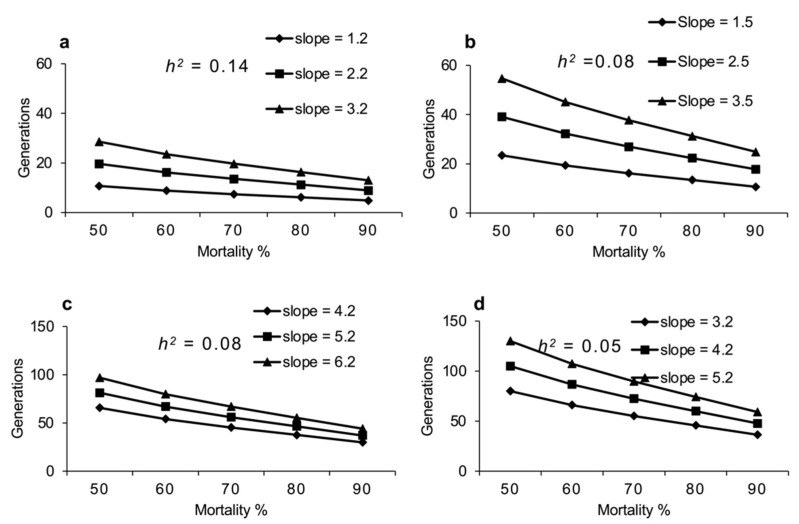
Effect of slope on number of generations of *D. suzukii* required for a 10-fold increase in LC_50_ of female exposed to spinosad (**a**), male exposed to spinosad (**b**), female exposed to malathion (**c**) and male exposed to malathion (**d**) at different selection intensities (i).

For any population of *D. suzukii*, the predicted response to selection in terms of number of generations needed for a 10-fold increase in LC_50_ (G) is directly proportional to slope assuming a constant heritability and inversely proportional to the heritability of resistance assuming a constant slope as shown in Figure 3 and Figure 4. For instance, if *h*^2^ = 0.14 (mean *h*^2^ of spinosad resistance in this study) and 90% of population was killed at each generation, 10-fold increase in LC_50_ of *D. suzukii* females would be expected in nine generations for spinosad at a slope of 2.2 (mean slope for spinosad in this study). However, everything else remaining the same, if the selection intensity decreases to a point where only 50% of the population is killed at each generation, 10-fold increase in LC_50_ of *D. suzukii* females would be expected in 20 generations for spinosad. Similarly, if *h*^2^ = 0.08 (mean *h*^2^ of malathion resistance in this study) and 90% of population was killed at each generation, it will take 37 generations to see 10-fold increase in LC_50_ of *D. suzukii* females at a slope of 5.2 (mean slope for malathion in this study). However, everything else remaining the same, if the selection intensity decreases to a point where only 50% of the population is killed at each generation, it will take 81 generations to see 10-fold increase in LC_50_ of *D. suzukii* females.

The response quotient is another parameter that can be used to compare the rates of resistance development in a population against different insecticides without reference to slope and assess the durability of various insecticides against a target pest. In the present study, the response quotient for resistance against spinosad was higher as compared to malathion. These results indicate that *D. suzukii* field populations will develop spinosad resistance at a faster rate as compared to malathion resistance. Thus, spinosad is more likely to lose efficacy in *D. suzukii* field populations in relatively shorter period of time compared to malathion.

Furthermore, in contrast with *h*^2^ estimates reported for male spinosad-selected *D. suzukii* in California organic orchards [14] and in male Oriental fruit moth [32], our study showed that *h*^2^ was generally higher in female compared with male, in both spinosad- and malathion-selected strains. It is presently unknown how the sex-specific effects of insecticides contribute to heritability of resistance in field populations of *D. suzukii*. Male flies are killed relatively faster than female cohorts exposed to similar concentration of insecticides, possibly because of small body size [23,33]. Autosomal inheritance of malathion and spinosad resistance has been reported in the Oriental fruit fly *Bactrocera dorsalis* [34], but female-linked spinosad resistance has also been documented in the house fly *Musca domestica* L. [35]. Future studies are needed to understand the role of sex-linkage in *D. suzukii* resistance to insecticides.

Resistance is more difficult to actualize when selection is started from inbred insect lines with little genetic variation than a more heterogeneous population with more genetic variation [26,30]. In our study, we found high and low slope estimates against malathion and spinosad selected populations, respectively. This finding may explain the slower rate of resistance development against malathion than spinosad. Furthermore, fly population from spinosad-sprayed fields may have had higher selection pressure as organic fruit farmers make multiple spinosad applications during the growing season due to limited chemistries to rotate unlike conventional farming systems, where growers can rotate between chemistries. The relatively quick response of this mixed population selected with spinosad in this study reflects a high risk for resistance development against spinosad in *D. suzukii* populations in the field. Unlike other major berry production regions in the United States [14], we have not seen any reports of control failures of *D. suzukii* associated to the use of spinosad in Georgia small fruit production systems. 

Our findings present an early warning for organic blueberry growers and pest managers that increased effort in resistance management strategies is needed to continue to benefit from the effectiveness of spinosad in management of *D. suzukii.* Furthermore, given that *D. suzukii* can go over 13 generations in a year in warmer regions of the United States [36], blueberry farmers should adhere to best management practices to continue to benefit from malathion as the slower rate of resistance development to malathion observed in this study is not an absence of resistance. Moreover, caution is needed in extrapolating results from a laboratory selection to field situation because every field population is different in terms of genetic variance and the exposure intensity, and laboratory data may not accurately predict resistance occurrence in actual field situations [37,38].

In conclusion, our study clearly showed that statistically significant levels of resistance to spinosad and malathion were developed in *D. suzukii* after only 10 and 11 generations of selection as compared to the unselected mixed population. This suggests that resistance alleles are present in the field populations even though control failures from spinosad and malathion applications have not been reported yet. The presence of resistance alleles coupled with extreme dependence on spinosad and malathion as control tools for *D. suzukii* infestations indicate that the risk of resistance development in *D. suzukii* field populations against malathion and spinosad is high. For an organism that completes a generation within 8–10 days at optimal temperature of around 25 °C, multiple generations can be exposed to repeated applications of malathion and spinosad based on their current used patterns to control *D. suzukii* in blueberries. Such repeated exposure to these insecticides further increases the likelihood of resistance development in field populations of *D. suzukii.* In similar situations, high levels of field-evolved resistance has been documented in other species including *Heliothis armigera*, *Bactrocera oleae*, and *Anthonomus grandis* [8,10,11]. It is therefore critical to continue to monitor *D. suzukii* field populations for resistance and at the same time proactively implement IRM strategies by rotating insecticides with different modes of action into *D. suzukii* season-long management programs [3,13,15]. Further studies should proactively determine resistance mechanisms such that scientifically based IRM programs are implemented to prolong the effective life of both insecticides extremely important for *D. suzukii* management in organic and conventional berry production systems in the United States. As *D. suzukii* adults can move between blueberry fields and potential wild hosts in the surrounding wooded areas, which may serve as untreated refuge, future studies should investigate the impact of such movement on rates of resistance development and persistence of resistance genes in field populations.

## Figures and Tables

**Table 1:** Effect of spinosad residue on adult male and female *D. suzukii* from spinosad selected (SPN-sel) and unselected susceptible mixed field population.

Selected Generation	Pop	Sex	Slope (±SE)	LC_50_ (ppm) (95% FL) ^†^	RR ^a^
	Unselected	Male	2.05 (0.38)	20.39 (8.78–41.59)	
F_3_	SPN-sel	Male	3.88 (1.14)	26.00 (19.00–36.93)	1.28 (0.25–0.67)
F_8_	SPN-sel	Male	1.97 (0.35)	60.45 (23.43–131.21)	2.96 (0.21–0.72)
F_10_	SPN-sel	Male	2.91 (0.54)	105.79 (71.11–153.15)	5.19 (0.15–0.44)
	Unselected	Female	2.27 (0.50)	22.14 (7.16–52.88)	
F_3_	SPN-sel	Female	1.96 (0.34)	50.72 (21.28–100.82)	2.29 (0.13–0.41)
F_8_	SPN-sel	Female	1.71 (0.31)	65.73 (25.30–164.18)	2.97 (0.18–0.68)
F_10_	SPN-sel	Female	2.47 (0.54)	167.25 (95.62–387.14)	7.55 (0.11–0.33)

^a^ Resistance ratio (RR) = LC_50_ of SPN-sel/LC_50_ of unselected male and female mixed population, ^†^ = toxicity is significant when 95% fiducial limits did not overlap with unselected population.

**Table 2:** Effect of malathion residue on adult male and female *D. suzukii* from malathion selected (Mal-sel) and unselected susceptible mixed field population.

Selected Generation	Pop	Sex	Slope (±SE)	LC_50_ (ppm) (95% FL) ^†^	RR ^a^
	Unselected	Male	3.92 (0.75)	2.72 (1.71–3.29)	
F_7_	Mal-sel	Male	3.76 (0.69)	3.23 (1.39–4.67)	1.19 (0.41–0.85)
F_9_	Mal-sel	Male	4.15 (0.83)	3.01 (1.53–4.16)	1.11 (0.47–1.05)
F_11_	Mal-sel	Male	5.72 (1.02)	5.49 (3.55–7.28)	2.02 (0.49–0.87)
	Unselected	Female	4.70 (1.03)	3.50 (2.23–4.55)	
F_7_	Mal-sel	Female	3.95 (0.74)	3.72 (2.07–5.02)	1.06 (0.58–1.13)
F_9_	Mal-sel	Female	4.71 (0.10)	3.69 (2.77–4.45)	1.05 (0.52–1.04)
F_11_	Mal-sel	Female	8.58 (1.64)	7.81 (6.61–9.12)	2.23 (0.48–0.78)

^a^ Resistance ratio (RR) = LC_50_ of Mal-sel/LC_50_ of unselected male and female mixed population; ^†^ = toxicity is significant when 95% fiducial limits did not overlap with unselected population.

**Table 3:** Estimation of response to selection (*R*) and selection differential (*S*) of spinosad-selected adult population of *D. suzukii*.

Selected Generation	Estimation of Response to Selection				Estimation of Selection Differential				
	Sex	Initial LC_50_ (95% FL)	Final LC_50_ (95% FL)	R	i	Initial Slope (±SE)	Final Slope (±SE)	*σ_p_*	s
F_3_	Male	20.39 (8.78–41.59)	26.00 (19.00–36.93)	0.01	1.74	2.05(0.38)	3.88 (1.14)	0.34	0.59
F_8_	Male	20.39 (8.78–41.59)	60.45 (23.43–131.21	0.07	1.76	2.05(0.38)	1.97 (0.35)	0.5	0.88
F_10_	Male	20.39 (8.78–41.59)	105.79 (71.11–153.15)	0.07	1.21	2.05(0.38)	2.91 (0.54)	0.4	0.49
F_3_	Female	22.14 (7.16–52.88)	50.72 (21.28–100.82)	0.12	1.45	2.27(0.50)	1.96 (0.34)	0.47	0.69
F_8_	Male	20.39 (8.78–41.59)	60.45 (23.43–131.21	0.07	1.76	2.05(0.38)	1.97 (0.35)	0.5	0.88
F_10_	Female	22.14 (7.16–52.88)	167.25 (95.62–387.14)	0.08	1.21	2.27(0.50)	2.47 (0.54)	0.42	0.51

**Table 4:** Estimation of response to selection (*R*) and selection differential (*S*) of malathion-selected adult population of *D. suzukii*.

Selected Generation	Estimation of Response to Selection				Estimation of Selection Differential				
	Sex	Initial LC50 (95% FL)	Final LC50 (95% FL)	R	i	Initial Slope (±SE)	Final Slope (±SE)	*σ_p_*	s
F_7_	Male	2.72 (1.71–3.29)	3.23 (1.39–4.67)	0.01	1.27	3.92 (0.75)	3.76 (0.69)	0.26	0.33
F_9_	Male	2.72 (1.71–3.29)	3.01 (1.53–4.16)	0.008	1.1	3.92 (0.75)	4.15 (0.83)	0.25	0.27
F_11_	Male	2.72 (1.71–3.29)	5.49 (3.55–7.28)	0.03	1.23	3.92 (0.75)	5.72 (1.02)	0.21	0.26
F_7_	Female	3.50 (2.23–4.55)	3.72 (2.07–5.02)	0.004	1.01	4.70 (1.03)	3.95 (0.74)	0.23	0.23
F_9_	Female	3.50 (2.23–4.55)	3.69 (2.77–4.45)	0.003	1.08	4.70 (1.03)	4.71 (0.10)	0.21	0.23
F_11_	Female	3.50 (2.23–4.55)	7.81 (6.61–9.12)	0.03	1.04	4.70 (1.03)	8.58 (1.64)	0.15	0.16
